# Early postnatal growth predictors of catch-up growth in term small-for-gestational-age infants: a nationwide propensity-score–matched study

**DOI:** 10.3389/fendo.2026.1757905

**Published:** 2026-03-12

**Authors:** Jinjoo Choi, Yun Jin Kim, Myoung-Jin Yoo, Jeesun Yoo, Ja-Hye Ahn, Yunsoo Choe, Seung Yang

**Affiliations:** 1 Department of Pediatrics, Hanyang University Hospital, Seoul, Republic of Korea; 2 Department of Pre-Medicine, College of Medicine, Hanyang University, Seoul, Republic of Korea; 3 Department of Pediatrics, Hanyang University Guri Hospital, Guri, Republic of Korea; 4 Department of Pediatrics, College of Medicine, Hanyang University, Seoul, Republic of Korea

**Keywords:** birth weight, breast feeding, growth, infant, small for gestational age

## Abstract

**Background:**

Infants born small for gestational age (SGA) are at risk for impaired growth and metabolic complications. However, the long-term effects of early feeding type on catch-up growth (CUG) in SGA infants remain controversial, and there have been no established anthropometric thresholds to predict later CUG.

**Methods:**

This population-based study analyzed full-term SGA infants (birth weight <3rd percentile) from the Korean National Health Screening Program (2007-2014). Feeding type was classified as exclusively breastfeeding or exclusively formula-feeding based on consistent parent-reported data through the first year. Infants receiving mixed breast- and formula-feeding or special formulas were excluded to ensure a clear comparison between distinct feeding modalities. CUG was defined as height > 3rd percentile by 42–48 months. Propensity-score matching (1:1) was applied for sex, birth weight, socioeconomic status, and residence.

**Results:**

After 1:1 propensity-score matching, a final cohort of 1,832 infants was analyzed. Formula-fed infants exhibited faster early growth, but intergroup differences diminished after 9–12 months, resulting in comparable CUG rates at four years of age. Feeding type was not significantly associated with CUG (adjusted odds ratio 1.38; 95% CI 0.76–2.51). In contrast, weight and height Z-scores at 4–6 months independently predicted later CUG (AUC 0.77 and 0.84, respectively).

**Conclusion:**

Early weight- and height-for-age Z-scores at 4–6 months are the key determinant of CUG at 4 years of age in term SGA infants. Despite slower initial growth, breastfeeding supports equivalent long-term outcomes and may represent a physiologically favorable trajectory. Growth monitoring at 4–6 months may help identify infants at risk of persistent growth failure and provide a basis for considering timely nutritional support.

## 1 Introduction

Children born small for gestational age (SGA) are at an increased risk of perinatal complications such as mortality, hypoglycemia, hypothermia compared with appropriate-for-gestational-age infants (1). They also tend to exhibit rapid pubertal progression, precocious adrenarche, and growth retardation later in life (2, 3). Many SGA infants experience catch-up growth (CUG), a compensatory postnatal acceleration that offsets intrauterine growth restriction (4). Although most full-term SGA infants achieve CUG within the first 12–24 months, approximately 10–15% fail to catch up and remain short into adulthood (5). Inadequate CUG during infancy has been associated with adverse neurocognitive outcomes and poorer psychosocial performance (6, 7). Therefore, identifying early predictors of inadequate CUG is critical for timely monitoring and intervention.

CUG in SGA infants typically occurs most rapidly during the first 3–6 months of life, a period in which breast milk or formula constitutes the predominant nutritional source (8). Prior findings on feeding type and CUG have been inconsistent, due to heterogeneity in study designs, small sample sizes, and short follow-up periods (9–13). Although formula feeding generally induces faster weight gain because of its higher protein and energy content (14–16), whether such early acceleration represents a favorable growth pattern in SGA infants remains debated (17). Early and excessive CUG may impair satiety regulation (18), which in turn may increase the risk of long-term cardiometabolic morbidities, including obesity, hypertension, diabetes, and hypercholesterolemia (19). Thus, achieving appropriate rather than excessive CUG is an important clinical goal.

Despite these concerns, few studies have established specific cutoff values for early anthropometric indices that can reliably predict subsequent CUG. Therefore, using a large nationwide dataset, this study aimed (i) to compare rates up to 4 years of age between breastfed and formula-fed term SGA infants using a propensity-score–matched design, and (ii) evaluate whether height and weight Z-scores obtained at the first round of the National Health Screening Program for Infants and Children (NHSPIC) (20)—a critical CUG period (4–6 months of age)—independently predict later CUG and determine their optimal cutoff values through receiver operating characteristic (ROC) analysis.

## 2 Materials and methods

### 2.1 Study design and data source

This study utilized data from the National Health Insurance Service (NHIS) database, a single-payer system covering approximately 98% of the Korean population. Since 2007, the NHIS has implemented the NHSPIC, which provides seven assessments for children under six. The NHSPIC evaluates nutritional status, anthropometry, and developmental milestones at 4–6, 9–12, 18–24, 30–36, 42–48, 54–60, and 66–71 months of age (20).

### 2.2 Study population

This population-based study included all eligible subjects meeting the inclusion criteria from the NHIS database during the study period (2007-2014). Sample size was not predetermined but was determined by applying rigorous inclusion and exclusion criteria to the entire national cohort. Among 2,780,166 children born between 2007 and 2014 who completed the 1st through 5th NHSPIC rounds, 135,193 were identified as SGA with birth weights below the 10th percentile, as defined by the Korean Pediatric Endocrine Society using the 2007 Korean National Growth Chart developed by the Korea Centers for Disease Control and Prevention (21, 22).

We applied the 3rd percentile birth-weight threshold to align with the Korean National Health Insurance eligibility criteria for growth hormone therapy (23). This threshold was also chosen to maintain consistency with our primary outcome of interest: the failure to achieve CUG, defined as remaining below the 3P in height at 42–48 months. This approach is supported by previous evidence (12), which confirms that postnatal growth trajectories do not significantly differ between SGA infants in the <3rd and <10th percentile groups. Because gestational age is not available in the NHIS database, the 3^rd^ percentile threshold for 37 weeks of gestation (2,290 g for girls and 2,380 g for boys) was applied to refine the SGA cohort. Children whose birth weights exceeded these thresholds were excluded from the analysis (21).

Diagnostic codes were extracted using the International Classification of Diseases, 10th Revision. Children were excluded if they:

1. were born preterm (ICD-10 P07);
2. had congenital or chromosomal anomalies (ICD-10 Q00–Q99);
3. had chronic pediatric disorders such as neoplasms (ICD-10 C), neurological disorders (ICD-10 G1/G7/G8), inflammatory bowel disease (ICD-10 K50/K51), chronic kidney disease (ICD-10 N18), or endocrine disorders (ICD-10 E2), including growth hormone deficiency (ICD-10 E23.0);
4. had extreme anthropometric Z-scores (> +3 or < –3) for weight or height at any screening round, which were defined as outliers to ensure data integrity;
5. had missing demographic information; or
6. consumed special, goat, soy, raw cow’s milk, iron-fortified formula, or mixed breast- and formula-feeding during the 1st or 2nd NHSPIC rounds.

**Figure 1:**
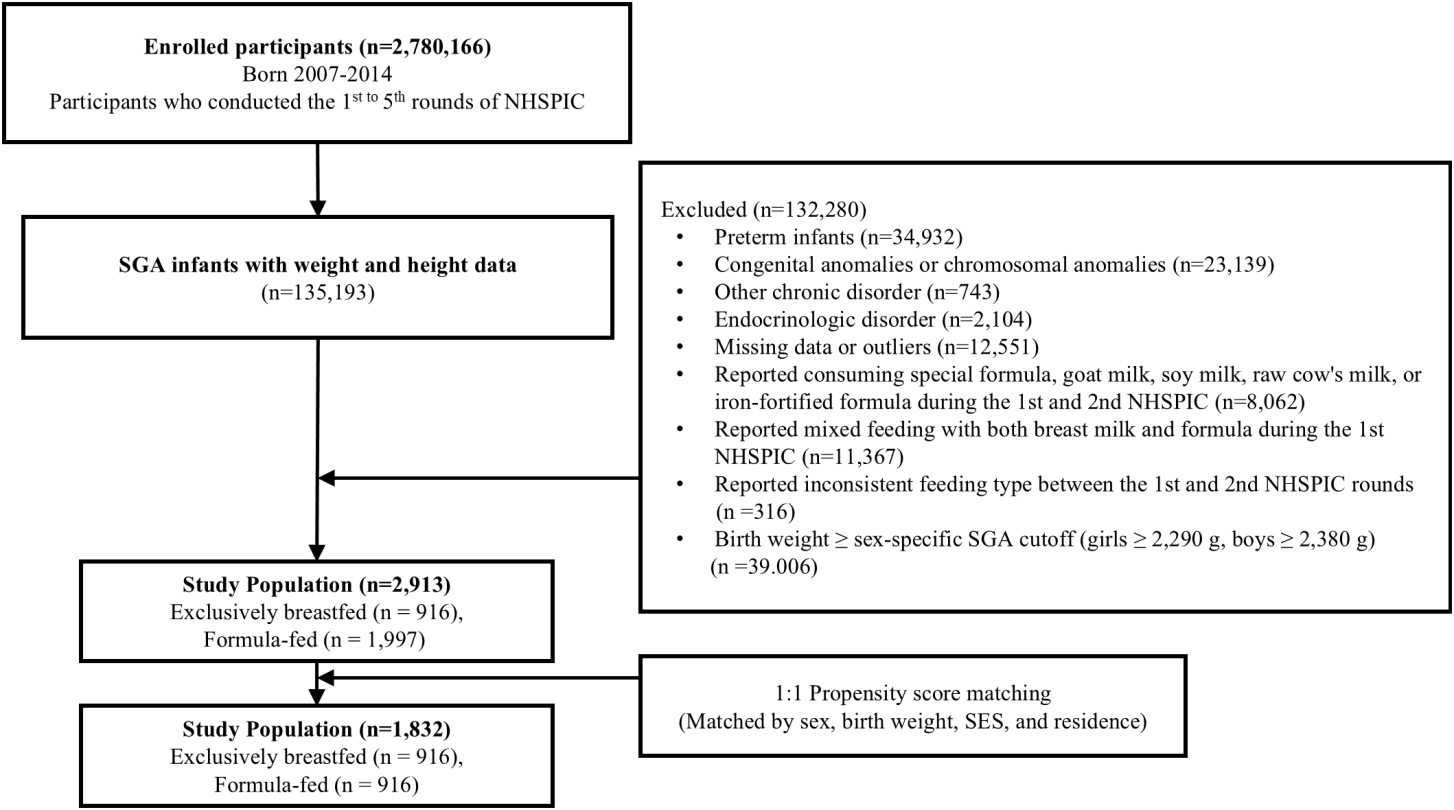
Flowchart of the study population selection process. The initial cohort consisted of 2,780,166 infants. After applying exclusion criteria, 2,913 term SGA infants were identified. Infants receiving mixed feeding or special formulas were excluded to ensure a clear comparison between exclusive feeding modalities. Subsequently, 1:1 propensity-score matching was performed, resulting in a final matched cohort of 1,832 infants. SGA, small for gestational age; NHSPIC, National Health Screening Program for Infants and Children.

After exclusions, 2,913 children (1,746 boys and 1,167 girls) were included for analysis. Subsequently, 1:1 propensity-score matching (PSM) was performed to balance baseline characteristics, resulting in a final matched cohort of 1,832 infants. Matching balance was verified by standardized mean differences (SMD). The selection flow diagram is presented in **Figure 1**.

### 2.3 Definitions

Feeding type was determined from parent-reported questionnaires at the 1st and 2nd NHSPIC assessments:

- Exclusively breastfed group: exclusively breastfed at both the 1st (4–6 months) and 2nd (9–12 months) rounds, indicating sustained exclusive breastfeeding for at least 9–12 months.
- Exclusively formula-fed group: exclusively formula-fed at both rounds.

Infants receiving mixed feeding (both breast milk and formula) or special formulas were excluded to reduce potential confounding effects and to focus on the divergent growth outcomes associated with exclusive feeding modalities. In South Korea, national health insurance covers growth hormone therapy for children born SGA who do not achieve CUG (height < 3rd percentile by age 4) (23). Accordingly, CUG was defined as attaining height ≥3rd percentile at the 5th NHSPIC round (42–48 months), based on the 2007 Korean National Growth Charts (22).

### 2.4 Anthropometric measurements

Weight and height were measured in real-time by trained medical professionals during each screening visit. Measurements were performed using standardized equipment at each participating healthcare facility following protocols established by the NHIS (20). Height was measured to the nearest 0.1 cm using an infantometer for children under 24 months and a stadiometer for those aged ≥2 years. Weight was measured using an electronic scale accurate to 100 g. Body mass index (BMI) was calculated as weight (kg) divided by height squared (m²). Height and weight Z-scores were derived from the 2007 Korean National Growth Chart reference (22).

### 2.5 Statistical analysis

Baseline demographic characteristics were summarized as number (%) for categorical variables or mean ± SD for continuous variables. The chi-square or Fisher’s exact test was used for categorical variables, and the Kruskal–Wallis test for continuous variables. Normality was assessed using the Anderson–Darling test.

To minimize baseline differences, 1:1 PSM) was performed between the breastfed and formula-fed groups, matching on sex, birth weight (per 100 g strata), socioeconomic status (SES), and residential area. Logistic regression was performed on the matched sample, and results were expressed as adjusted odds ratios (aORs) with 95% confidence intervals (CIs).

SES was inferred from health insurance premiums and categorized into quartiles. Birth weight was obtained from parent-reported questionnaires at the 1st NHSPIC round. BMI at the 5th round was included in multivariable models to examine whether current adiposity influences CUG.

Receiver operating characteristic (ROC) analysis was used to determine optimal cutoff values for height and weight Z-scores at 4–6 months. The primary performance metric was the area under the ROC curve (AUC). Because CUG outcomes were highly imbalanced, supplementary performance metrics—including the area under the precision–recall curve (AUPRC), Brier score, and Akaike information criterion (AIC)—were evaluated to assess discrimination, calibration, and relative model fit.

Statistical significance was defined as *p* < 0.05. All analyses were conducted using SAS version 9.4 (SAS Institute Inc., Cary, NC, USA).

### 2.6 Ethics statement

This study used anonymized secondary data provided by the NHIS. All personal identifiers were removed prior to data access, and investigators had access only to encrypted identification numbers. Data were analyzed within a secure server environment and were not downloaded or redistributed. The requirement for informed consent was waived because no direct interaction with participants occurred and no identifiable information was used. The study protocol was approved by the Institutional Review Board of Hanyang University Hospital (IRB No. 2024-01-012-001).

## 3 Result

### 3.1 Comparison of growth trajectories between breastfed and formula-fed infants

The baseline demographic characteristics of the study population before and after PSM are summarized in **Table 1**. Among 2,913 infants, 31.4% (n = 916) were breastfed and 68.6% (n = 1,997) were formula-fed. The median birth weight was 2.3 kg (interquartile range [IQR], 2.3–2.4 kg). Before matching, 98.7% of term SGA infants had achieved CUG by four years of age, and there were no significant differences between the two groups in sex distribution, birth weight, socioeconomic status, or residential area (all *p* > 0.05).

**Table 1:** Comparison of baseline characteristics before and after propensity-score matching^a^.

Variable	Before Matching (Full cohort, N = 2,913)		After Matching (Matched cohort, N = 1,832)	
	Breastfed (n = 916)	Formula-fed (n = 1,997)	Breastfed (n = 916)	Formula-fed (n = 916)
Birth outcomes				
Sex (Female), %	41.92	39.21	41.92	41.92
Birth weight (kg), median	2.3	2.3	2.3	2.3
Socioeconomics^b^, %				
SES: 4th (highest)	22.05	25.59	22.05	22.05
SES: 1st (lowest)	11.24	10.52	11.24	11.24
Residence (Rural)^c^, %	53.93	55.83	53.93	53.93

Feeding groups were classified based on exclusive feeding status at both the 1st (4–6 months) and 2nd (9–12 months) NHSPIC rounds. Infants with mixed feeding or special formulas were excluded. Data are expressed as percentage (%) or median. ^a^Propensity scores were estimated using sex, birth weight, SES, and residence to balance the groups. Successful covariate balance was confirmed as all standardized mean differences (SMDs) were < 0.1 after matching (see **Supplementary Figure 1**). ^b^Only the highest (4th) and lowest (1st) quartiles for SES are presented for clarity; all quartiles were included in the propensity-score matching. ^c^Urban includes metropolitan and city areas; Rural includes country areas.

SES, socioeconomic status.

After 1:1 PSM based on sex, birth weight, socioeconomic status, and residential area, covariate balance was confirmed with all SMDs < 0.1 and non-significant p-values (**Supplementary Figure 1**).

**Figure 2:**
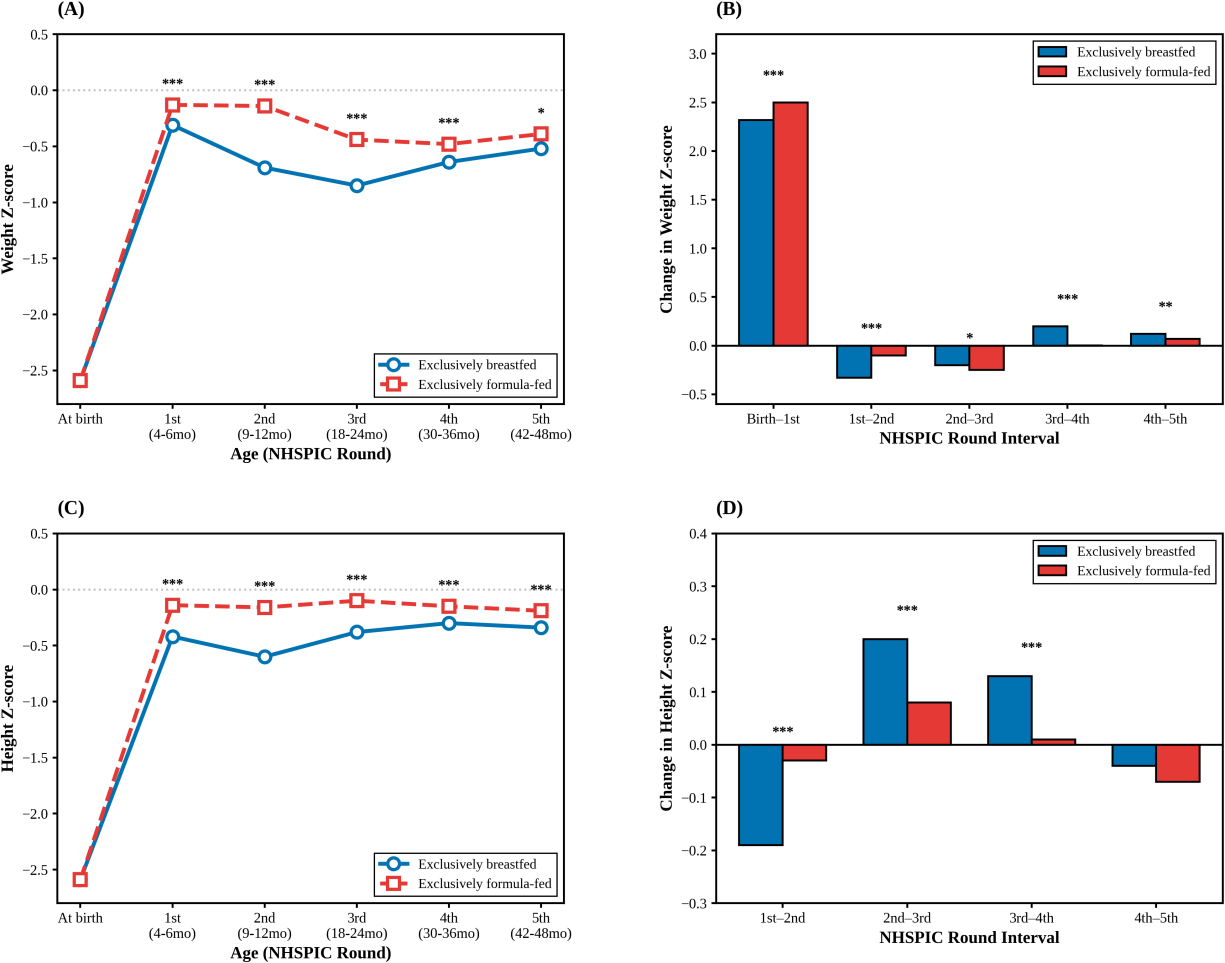
Longitudinal postnatal growth trajectories of term small-for-gestational-age (SGA) infants by feeding type. **(A, C)** Mean weight and height Z-scores from birth to 4 years of age. **(B, D)** Incremental changes in Z-scores between consecutive NHSPIC screening rounds (e.g., “Birth–1st” indicates the change from birth to the 4–6 month screening). The solid blue lines represent the exclusively breastfed group, and the dashed red lines represent the exclusively formula-fed group. *p < 0.05, **p < 0.01, ***p < 0.001 for comparisons between the two groups at each time point. NHSPIC, National Health Screening Program for Infants and Children.

**Figure 2** illustrates longitudinal changes in growth parameters between the two feeding groups from birth to the fifth NHSPIC round after PSM. At birth, both groups showed similarly low weight Z-scores, consistent with SGA status. A rapid catch-up in weight occurred during the first NHSPIC round, indicating that the magnitude of CUG was greatest between birth and the first visit, followed by stabilization (**Figure 2A**).

Throughout follow-up, formula-fed infants exhibited significantly higher weight and height Z-scores than breastfed infants (*p* < 0.001; **Figures 2A, C**; **Supplementary Table 1**). However, after the second NHSPIC round, breastfed infants demonstrated a steeper upward trajectory, and the intergroup gap in both weight and height progressively narrowed over time (**Figures 2B, D**; **Supplementary Table 2**).

### 3.2 Predictors and early indicators of catch-up growth

**Table 2** presents the logistic regression analysis of predictors of CUG after PSM. Feeding type (exclusive breastfeeding vs. exclusive formula feeding) was not significantly associated with achieving CUG (aOR, 1.38; 95% CI, 0.76–2.51; p = 0.29). In contrast, the weight Z-score measured at the first NHSPIC round was a strong and independent predictor of subsequent CUG (aOR, 2.31; 95% CI, 1.62–3.30; p < 0.0001), indicating that infants with higher early postnatal weight Z-scores were more likely to achieve CUG by four years of age. Sex, birth weight, SES, and residential area were not significantly associated with CUG.

**Table 2:** Logistic regression analysis of predictors for catch-up growth success at 4 years of age^a^.

Variable	OR (95% CI)*	p-value
Feeding type		
Exclusively breastfed	Reference	
Exclusively formula-fed	1.38(0.76-2.51)	0.29
Early Growth Status (4–6 months)		
1^st^ NHSPIC Weight Z-score	2.31(1.62-3.30)	<.0001
Demographics & Socioeconomics		
Sex (Female vs. Male)	1.19(0.65-2.20)	0.57
Birth weight (kg)	0.03(<0.001 – 2.21)	0.11
SES (1st vs. 4th quartile)^b^	0.50 (0.16-1.58)	0.24
Residence (Rural vs. Urban)	0.58(0.31-1.08)	0.09
Adiposity at 4 years		
BMI (5^th^ NHSPIC)	1.13(0.87-1.46)	0.35

Catch-up growth (CUG) was defined as achieving height ≥3rd percentile at 42–48 months (5th NHSPIC round) based on the 2007 Korean National Growth Charts. Feeding groups were classified based on exclusive feeding status at both the 1st (4–6 months) and 2nd (9–12 months) NHSPIC rounds. Data are presented after 1:1 propensity-score matching. Sample size (N = 1,832) represents the matched cohort. ^a^Propensity scores were estimated using sex, birth weight, SES, and residence as covariates to balance the feeding groups. ^b^Only the highest (4th) and lowest (1st) quartiles for SES are presented for clarity, although all quartiles were included in the model. *Interpretation: An OR > 1 indicates a higher likelihood of achieving CUG (height ≥ 3rd percentile) by age 4.

CUG, catch-up growth; OR, odds ratio; CI, confidence interval; SES, socioeconomic status; NHSPIC, National Health Screening Program for Infants and Children; BMI, body mass index.

**Figure 3:**
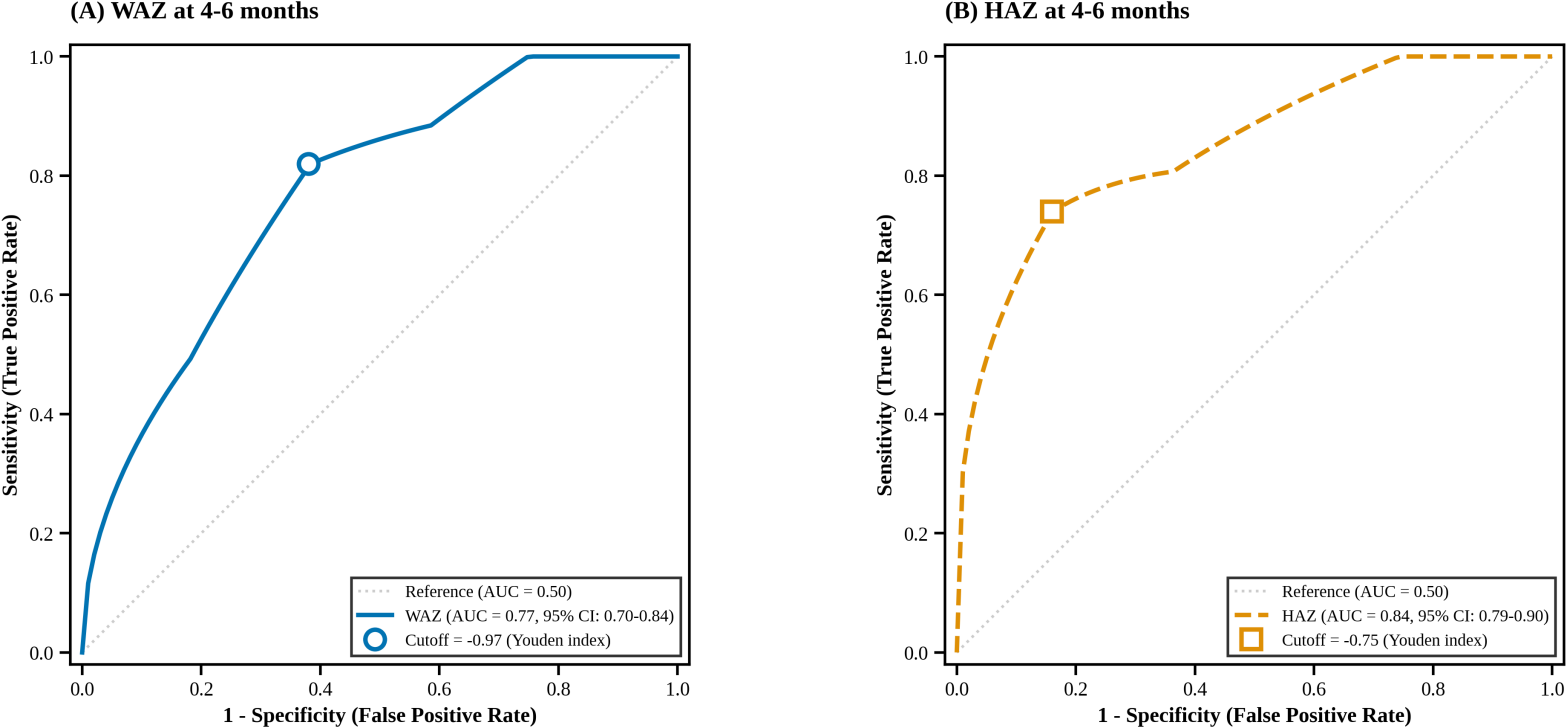
Receiver operating characteristic (ROC) curves of early growth indicators for predicting catch-up growth at 4 years in term SGA infants. **(A)** Weight-for-age Z-score (WAZ) at the first NHSPIC screening (4–6 months) shown as a blue solid line (AUC = 0.77, 95% CI: 0.70-0.84). **(B)** Height-for-age Z-score (HAZ) at the first NHSPIC screening (4–6 months) shown as an orange dashed line (AUC = 0.84, 95% CI: 0.79-0.90). The gray diagonal dotted line represents the reference for a random classifier (AUC = 0.50). Hollow markers indicate optimal cutoff points determined using the Youden index: -0.97 for WAZ (sensitivity 0.82, specificity 0.62) and -0.75 for HAZ (sensitivity 0.74, specificity 0.84). Detailed performance metrics are provided in **Table 3** and **Supplementary Table 3**. AUC, area under the curve; CI, confidence interval; HAZ, height-for-age Z-score; NHSPIC, National Health Screening Program for Infants and Children; ROC, receiver operating characteristic; SGA, small for gestational age; WAZ, weight-for-age Z-score.

Given the strong predictive value of the first-round anthropometric indices, ROC analysis was conducted to evaluate the discriminative performance of early weight and height Z-scores (**Table 3**, **Figure 3**). Both parameters demonstrated meaningful predictive accuracy for subsequent CUG, with AUC values of 0.77 for weight and 0.84 for height (**Figures 3A, B**). Corresponding cutoff values, sensitivities, and specificities are summarized in **Table 3**.

**Table 3:** Predictive performance of early growth indicators for catch-up growth at 4 years in term SGA infants^a^.

Model	Predictor	OR (95% CI)	AUC (95% CI)	Cutoff	Sensitivity	Specificity
M1	1^st^ NHSPIC Weight Z-score	2.31(1.62-3.30)	0.77(0.70-0.84)	-0.97	0.82	0.62
M2	1^st^ NHSPIC Height Z-score	2.74(1.87-4.00)	0.84(0.79-0.90)	-0.75	0.74	0.84

Catch-up growth (CUG) was defined as achieving height ≥3rd percentile at 42–48 months (5th NHSPIC round) based on the 2007 Korean National Growth Charts. Each model included one anthropometric predictor from the first screening round (4–6 months of age) to compare its individual predictive performance. Analysis was performed on 1,832 propensity-score matched infants. ^a^The optimal cutoff values were determined using the Youden index (sensitivity + specificity − 1). Supplementary performance metrics are provided in **Supplementary Table 3**.

AUC, area under the curve; CI, confidence interval; NHSPIC, National Health Screening Program for Infants and Children; OR, odds ratio; SGA, small for gestational age.

## 4 Discussion

### 4.1 Summary of key findings

In this nationwide cohort of term SGA infants, early postnatal anthropometric status at 4–6 months was the strongest predictor of achieving CUG by four years of age. Although formula-fed infants showed faster early growth, this difference diminished after the first year, resulting in comparable CUG rates between feeding groups. These findings indicate that early growth adequacy, not feeding type, is the primary determinant of later CUG.

### 4.2 Early feeding and CUG

Prior studies have reported inconsistent results regarding whether breastfeeding or formula feeding better promotes CUG in term SGA infants. Some reported that breastfeeding facilitated CUG (11, 13, 24, 25), whereas others observed more rapid early CUG among formula-fed infants (11) (15). Such discrepancies likely reflect heterogeneity in study populations, definitions of CUG, and observation periods.

Our findings align with recent Korean data (12), which showed no significant difference in CUG success at 6 months across different feeding types. We further extend this evidence by demonstrating that this lack of association persists into early childhood (42–48 months).

The faster initial weight gain seen in formula-fed infants in our study can be interpreted through the “early protein hypothesis” (16), suggesting that the higher protein content in formula stimulates insulin-related growth factor. However, consistent with recent evidence from the 3D Cohort (15) and Giulia et al. (9), our study demonstrated that breastfeeding—despite slower early growth—provides a protective effect against unhealthy rapid weight gain while ensuring long-term growth outcomes. Thus, our findings reaffirm the adequacy and safety of breast milk as the primary nutritional source for term SGA infants.

### 4.3 Early indicators and clinical implications

The first several postnatal months represent a critical window for growth recovery. In our cohort, weight-for-age Z-score (WAZ) and Height-for-age Z-score (HAZ) at 4–6 months independently predicted later CUG, enabling ROC-derived thresholds for early risk stratification.

These results align with Bouferoua et al. (24), who reported that failure to achieve CUG by six months was associated with persistent growth failure. Furthermore, our findings are supported by previous Korean data showing that the majority of SGA infants achieve significant CUG within the first 6 months of life (12). Similarly, large-scale Korean nationwide data reported that WAZ and HAZ at 4–6 months serve as critical predictors of short stature at four years in low-birth-weight infants (26). Together, this evidence highlights that early growth reflects intrinsic potential and is informative for later height.

Clinically, infants who fall below these thresholds may benefit from closer monitoring and nutritional assessment. Healthcare providers should consider evaluating milk intake adequacy (e.g., breastfeeding technique or formula volume), and providing timely guidance on the complementary feeding practices (6–12 months) to support healthy growth trajectories.

### 4.4 Metabolic implications

Rapid postnatal weight gain in SGA infants has been associated with higher risks of obesity, metabolic syndrome, and earlier puberty (16, 19, 27, 28). Recent large-scale Korean data further emphasize this by demonstrating a U-shaped relationship between weight status at age 2 and neurodevelopmental outcomes at age 6; both underweight (WAZ < 10th percentile) and overweight (WAZ ≥ 85th percentile) infants faced increased risks of suboptimal neurodevelopment. Moreover, infants with excessive weight gain by age 2 showed a 14-fold increased risk of obesity by age 6, with prevalence escalating sharply between ages 3 and 4 (7). In this context, the slower but steady catch-up pattern observed in exclusively breastfed infants may represent a physiologic and metabolically favorable trajectory that achieves ‘moderate’ CUG, thereby optimizing neurodevelopmental potential while minimizing the long-term risk of obesity. Further prospective longitudinal studies are needed to define optimal CUG rates that balance height potential with long-term cardiometabolic safety beyond early childhood.

### 4.5 High rate of catch-up growth

A notable finding is the exceptionally high CUG rate (98.7%), exceeding previous Korean estimates (29). Improved nutritional awareness, early screening, and exclusion of preterm or chronically ill infants may contribute to these favorable outcomes. Nonetheless, 1–2% of term SGA infants still fail to achieve adequate CUG, underscoring the importance of early surveillance using the thresholds proposed in this study.

### 4.6 Strengths and limitations

Major strengths include the large population-based cohort and the use of PSM, which minimized selection bias. Limitations include lack of gestational age and birth-length data, reliance on parent-reported feeding practices, and incomplete information on complementary feeding, specific formula types, brand-specific variations, and parental anthropometrics. Additionally, stringent exclusion criteria—while necessary to ensure a homogeneous cohort—may limit generalizability to SGA infants with comorbidities or mixed feeding patterns. Future studies incorporating these factors and extending follow-up into adolescence are warranted.

## Conclusion

Early anthropometric status at 4–6 months is a powerful predictor of later CUG in term SGA infants. Feeding type did not influence the likelihood of achieving CUG, supporting breastfeeding as a safe and adequate nutrition source. The ROC-derived cutoff values presented here can assist clinicians in early risk stratification and targeted monitoring. Further research examining maternal factors, feeding duration, and metabolic outcomes is needed to refine early growth management strategies.

## Data Availability

The datasets presented in this article are not readily available because The dataset used in this study is derived from the National Health Insurance Service (NHIS) database and the National Health Screening Program for Infants and Children (NHSPIC). These data are subject to strict legal and institutional restrictions and cannot be publicly shared or redistributed. Access is permitted only through the NHIS data access system upon institutional approval. Requests to access the datasets should be directed to National Health Insurance Service (NHIS) Data Request Portal: https://nhiss.nhis.or.kr.
