# Supplementary material for: Early postnatal growth predictors of catch-up growth in term small-for-gestational-age infants: a nationwide propensity-score–matched study
Source: Front Endocrinol (Lausanne). 2026 Mar 12;17:1757905. doi: 10.3389/fendo.2026.1757905 (PMC13017244; doi:10.3389/fendo.2026.1757905)
Supplement: Supplementary file 1 [file DataSheet1.docx]

Supplementary Material

**Supplementary Figure 1**


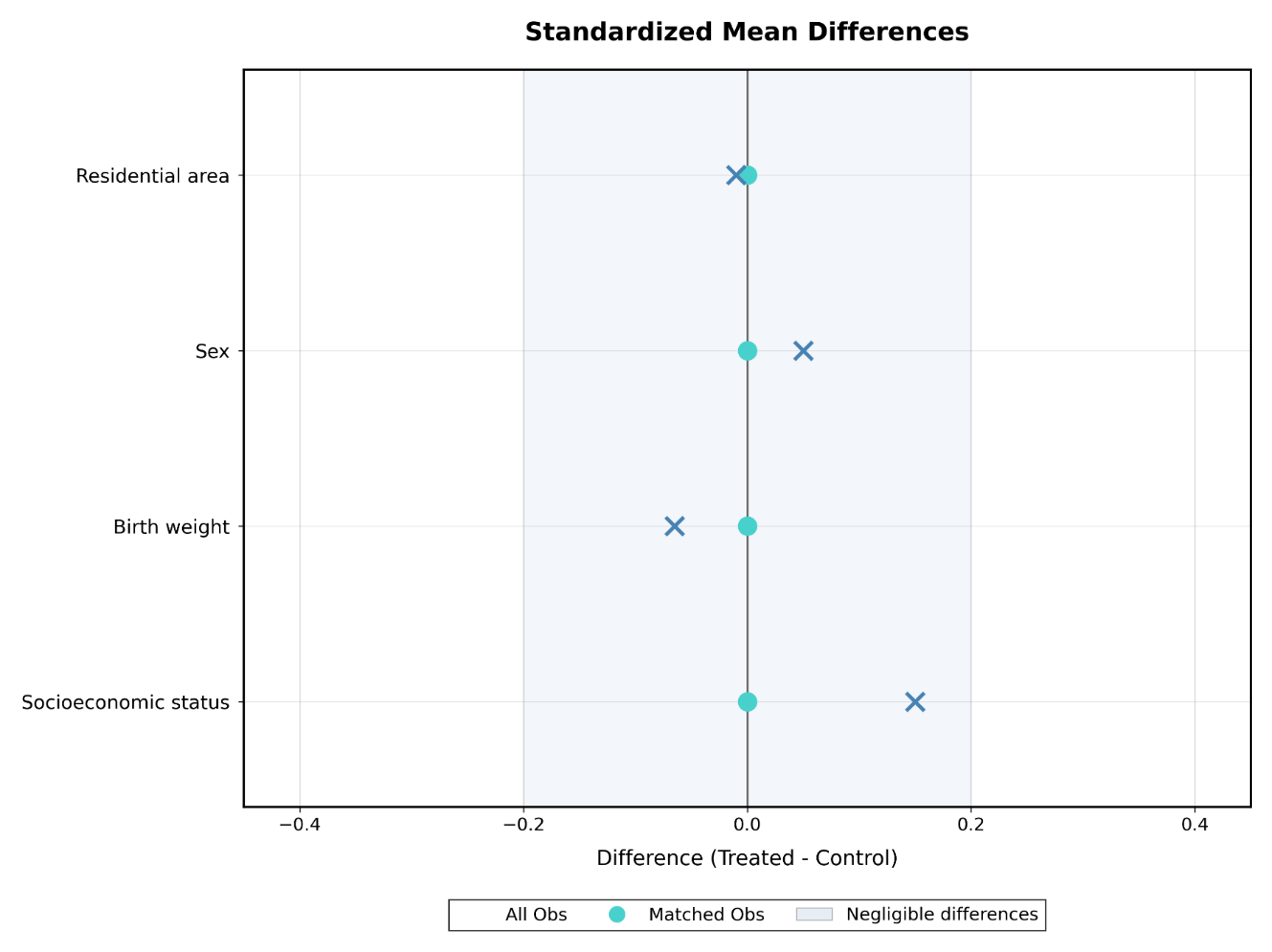


**Supplementary Figure 1.** **Standardized mean differences (SMDs) before and after propensity score matching (PSM).**
Standardized mean differences of baseline covariates (sex, birth weight, socioeconomic status, and residential area) were plotted before matching (blue crosses) and after matching (green circles). The shaded area (|SMD| < 0.1) indicates negligible imbalance, confirming that adequate covariate balance was achieved after matching.

**Supplementary table 1.** Comparison of height and weight Z-scores between breastfed and formula-fed infants after propensity-score matching. Data are expressed as median (interquartile range). BMI was calculated from height and weight measured at the 5th NHSPIC round.

|  | Total (n=1,832) | Only breastfed (n=916) | Only formula milk fed (n=916) | P-value |
| --- | --- | --- | --- | --- |
| At birth |  |  |  |  |
| Weight z-score | -2.59(-2.71 – -2.40) | -2.59(-2.71 – -2.40) | -2.59(-2.71 – -2.40) | 1 |
| 1^st^ NHSPIC |  |  |  |  |
| Height z-score | -0.26(-0.80 – 0.25) | -0.42(-0.92 – 0.17) | -0.14(-0.65 – 0.36) | <.0001 |
| Weight z-score | -0.19(-0.77– 0.41) | -0.31(-0.87– 0.31) | -0.13(-0.68– 0.53) | <.0001 |
| 2^nd^ NHSPIC |  |  |  |  |
| Height z-score | -0.39(-0.95 – 0.19) | -0.60(-1.10 – -0.05) | -0.16(-0.75 – 0.33) | <.0001 |
| Weight z-score | -0.41(-1.04 – 0.19) | -0.69(-1.24 – -0.09) | -0.14(-0.75 – -0.42) | <.0001 |
| 3^rd^ NHSPIC |  |  |  |  |
| Height z-score | -0.26(-0.83 – 0.26) | -0.38(-0.92 – 0.09) | -0.10(-0.70 – 0.40) | <.0001 |
| Weight z-score | -0.68(-1.22 – -0.03) | -0.85(-1.34 – -0.32) | -0.44(-1.05 – 0.20) | <.0001 |
| 4th NHSPIC |  |  |  |  |
| Height z-score | -0.22(-0.69 – 0.27) | -0.30(-0.78 – 0.15) | -0.15(-0.59 – 0.38) | <.0001 |
| Weight z-score | -0.56(-1.15 – 0.07) | -0.64(-1.24 – -0.05) | -0.48(-1.07 – 0.18) | <.0001 |
| 5th NHSPIC |  |  |  |  |
| Height z-score | -0.27 ( -0.83 – 0.26) | -0.34 (-0.90 – 0.19) | -0.19 (-0.78 – 0.36) | 0.0001 |
| Weight z-score | -0.45(-1.06 – 0.20) | -0.52 (-1.09 – 0.16) | -0.39 (-1.01 – 0.26) | 0.017 |
| BMI (kg/m²) | 15.31(14.61-16.12) | 15.27(14.60-16.14) | 15.33(14.61-16.11) | 0.7005 |

Data are expressed as median (Q1–Q3); P-values were calculated using the Mann–Whitney U test after propensity-score matching; Abbreviation: NHSPIC, National Health Screening Program for Infants and Children; BMI, body mass index; CUG, catch-up growth

**Supplementary table 2.** Comparison of Height and Weight Z-Scores Between Breastfed and Formula-fed Infants After Propensity Score Matching

|  | **Total (n=1,832)** | **Only breastfed (n=916)** | **Only formula milk fed (n=916)** | | **P-value** |
| --- | --- | --- | --- | --- | --- |
| **Change in weight Z-scores**† **(median, Q1-Q3)** |  |  |  | |  |
| Birth-1^st^ round | 2.40(1.84 – 3.01) | 2.32(1.74 – 2.92) | 2.50(1.95 – 3.13) | <.0001 | |
| 1^st^ – 2^nd^ round | -0.22(-0.59 – 0.14) | -0.33(-0.72 – -0.01) | -0.10(-0.46 – 0.27) | <.0001 | |
| 2^nd^ – 3^rd^ round | -0.23(-0.59 – 0.12) | -0.20(-0.58 – 0.16) | -0.25(-0.59 – 0.07) | 0.0157 | |
| 3^rd^ – 4^th^ round | 0.10(-0.23 – 0.40) | 0.20(-0.09 – 0.49) | 0.003(-0.33 – 0.29) | <.0001 | |
| 4^th^ – 5^th^ round | 0.10(-0.19 – 0.39) | 0.12(-0.18 – 0.42) | 0.07(-0.22 – 0.36) | 0.0031 | |
| **Change in height Z-scores**† **(median, Q1-Q3)** |  |  |  |  | |
| 1^st^ – 2^nd^ round | -0.11(-0.54 – 0.30) | -0.19(-0.62 – 0.21) | -0.03(-0.45 – 0.37) | <.0001 | |
| 2^nd^ – 3^rd^ round | 0.14(-0.24 – 0.46) | 0.20(-0.19 – 0.52) | 0.08(-0.28 – 0.42) | 0.0002 | |
| 3^rd^ – 4^th^ round | 0.06(-0.23 – 0.35) | 0.13(-0.18 – 0.42) | 0.01(-0.26 – 0.28) | <.0001 | |
| 4^th^ – 5^th^ round | -0.05(-0.30 – 0.16) | -0.04(-0.29 – 0.18) | -0.07(-0.30 – 0.14) | 0.1232 | |

Data are expressed as median (Q1–Q3). †Z-score changes were calculated as differences between consecutive NHSPIC rounds (birth–1st, 1st–2nd, 2nd–3rd, 3rd–4th, and 4th–5th). P-values were calculated using the Mann–Whitney U test. Abbreviation: NHSPIC, National Health Screening Program for Infants and Children.

**Supplementary table 3. Additional predictive performance metrics for anthropometric Z-score models from the first NHSPIC round.**

NHSPIC, National Health Screening Program for Infants and Children; AUPRC, Area Under the Precision-Recall Curve; AIC, Akaike information criterion.

| **Model** | **Predictor** | **AUPUC** | **Youden index** | **Brier** | **AIC** |
| --- | --- | --- | --- | --- | --- |
| M1 | 1^st^ NHSPIC Weight z-score | 0.99 | 0.44 | 0.02 | 398.48 |
| M2 | 1^st^ NHSPIC Height z-score | 0.99 | 0.59 | 0.02 | 392.66 |
